# Supplementary material for: Synthesis, Biological Evaluation and Computational Studies of New Hydrazide Derivatives Containing 1,3,4-Oxadiazole as Antitubercular Agents
Source: Int J Mol Sci. 2022 Dec 4;23(23):15295. doi: 10.3390/ijms232315295 (PMC9735621; doi:10.3390/ijms232315295)
Supplement: Supplementary file 1 [file ijms-23-15295-s001.zip › ijms-2015160-supplementary.pdf]

Supporting Information

Synthesis, biological evaluation and computational studies of new hydrazide derivatives containing 1,3,4-oxadiazole as antitubercular agents

Daniele Zampieri[1*], Sara Fortuna[2], Maurizio Romano[3], Alessandro De Logu[4], Gianluigi Cabiddu[4], Adriana Sanna[5] and Maria Grazia Mamolo[1]

[1]*Department of Chemistry and Pharmaceutical Sciences, Piazzale Europa 1, University of Trieste, 34127 Trieste, Italy.*
[2]*Italian Institute of Technology (IIT), Via E. Melen, 83, I-16152 Genova, Italy.*
[3]*Department of Life Sciences, Via Valerio 28/1, University of Trieste, 34127 Trieste, Italy*
[4]*Department of Life and Environmental Sciences, University of Cagliari, Cittadella Universitaria di Monserrato, 09042, Monserrato (Cagliari), Italy.*
[5]*Department of Public Health, Clinical and Molecular Medicine, University of Cagliari Cittadella Universitaria di Monserrato, 09042, Monserrato (Cagliari), Italy.*

[*]Correspondance: dzampieri@units.it

Table of Contents

1. Experimental data

   - Spectral data page 2
   - Cytotoxicity study page 20

NMR Spectra

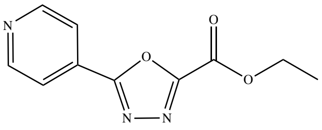

2a

Solvent: $CDCl_3$

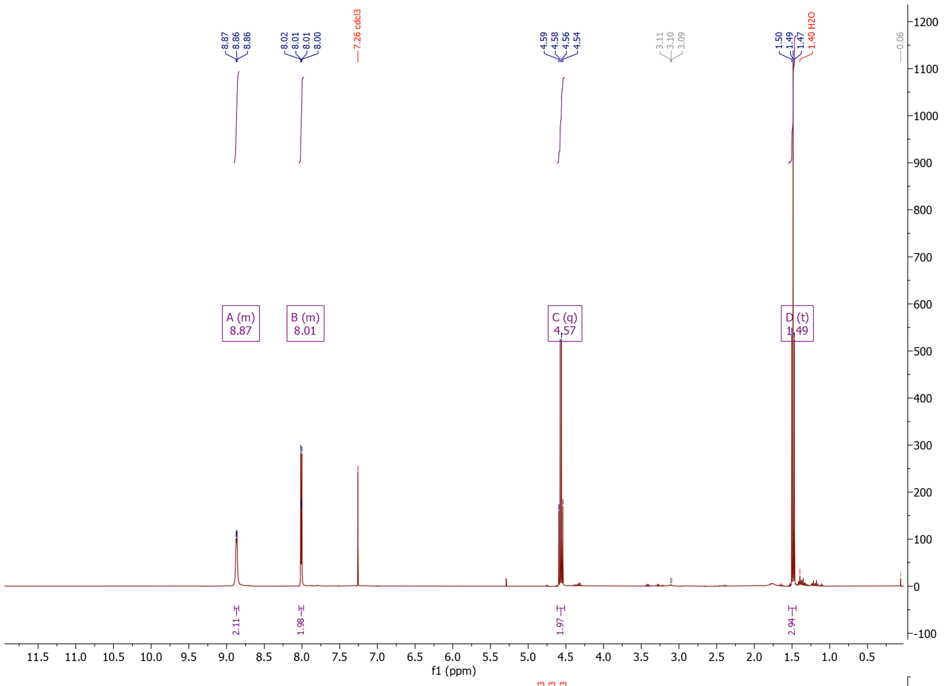

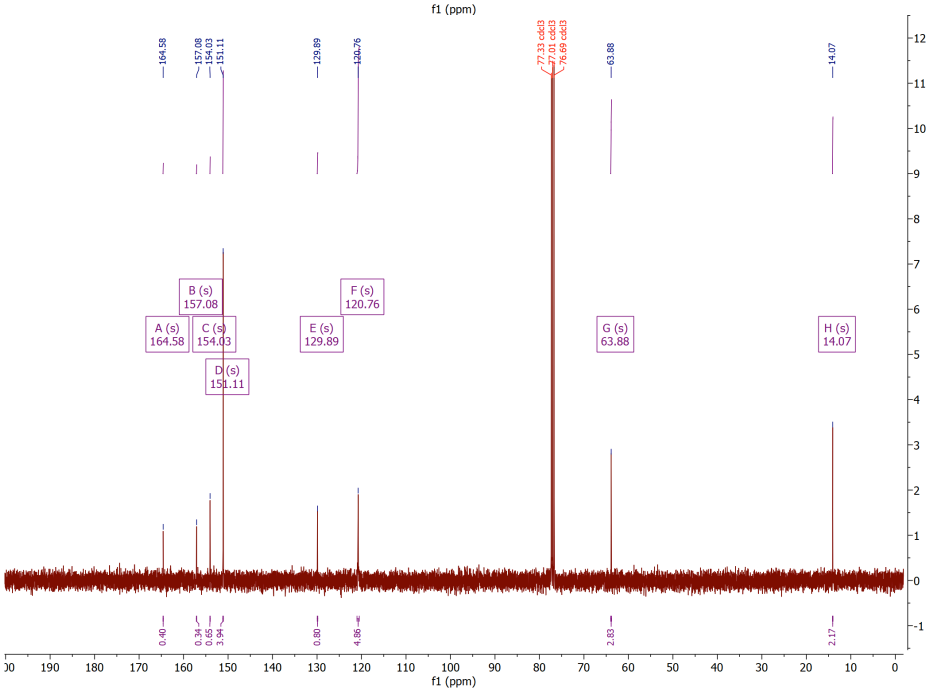

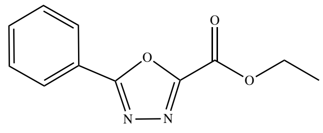

2b

Solvent: $CDCl_3$

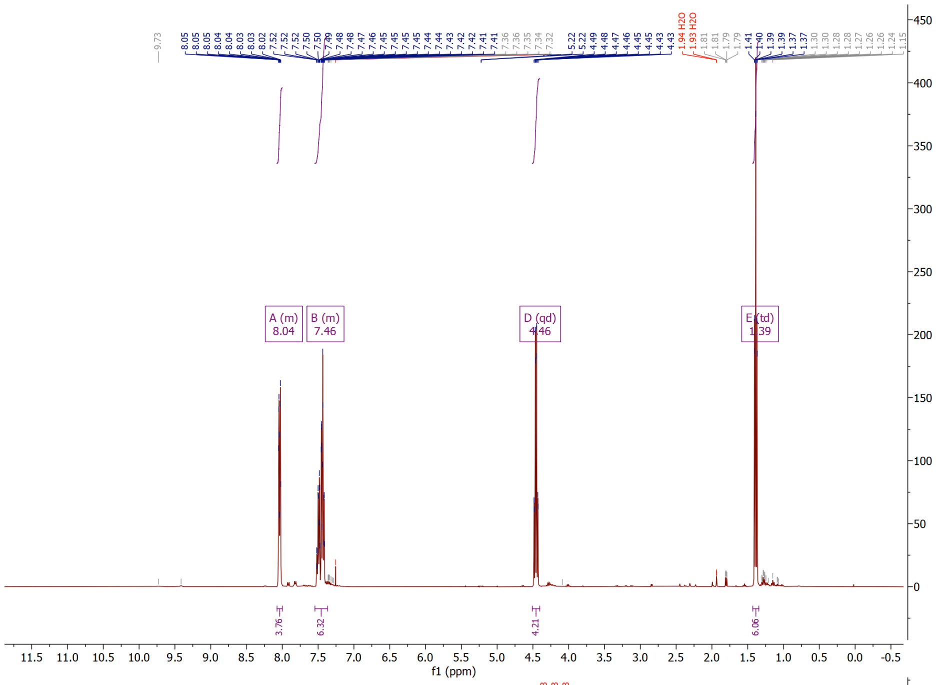

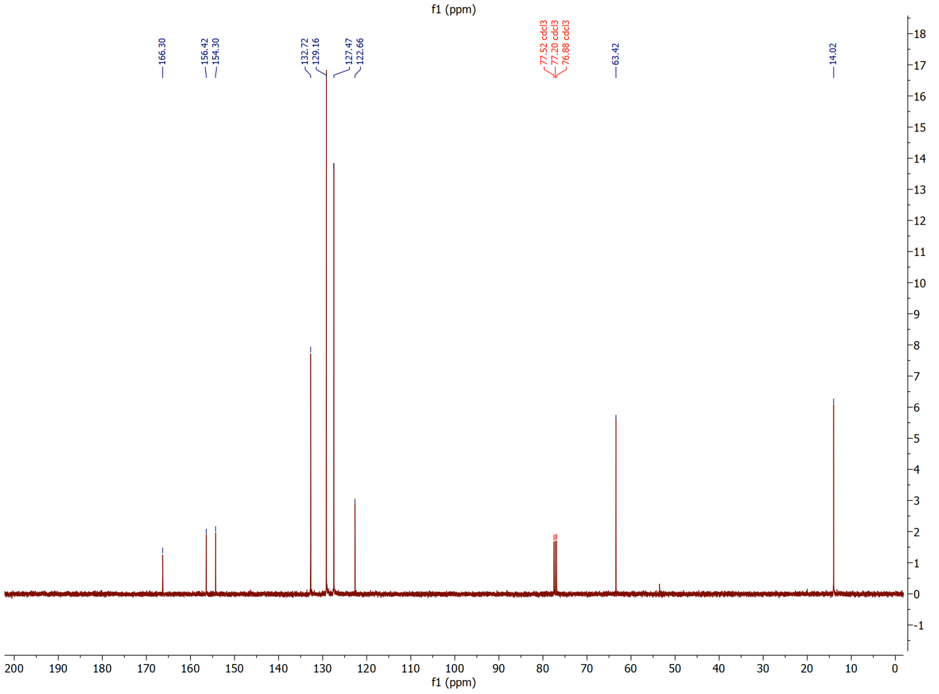

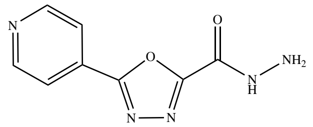

3a

Solvent: DMSO-d6

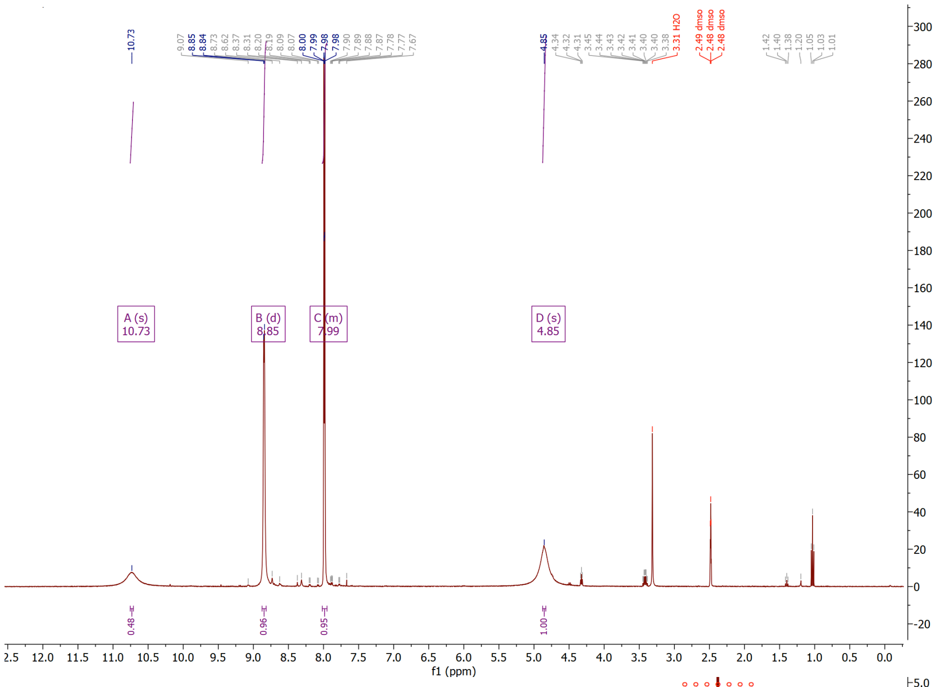

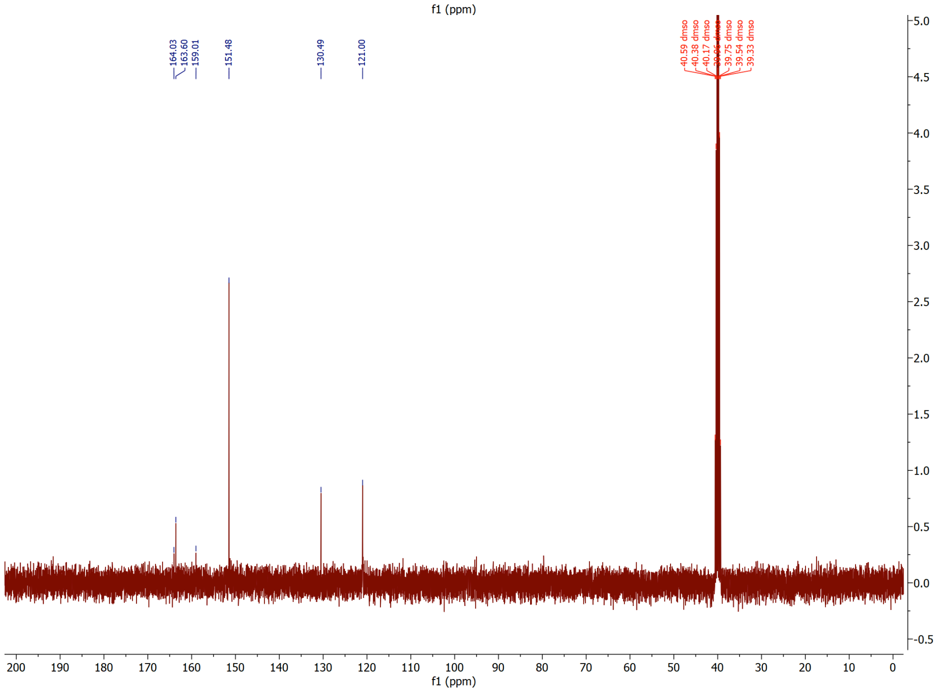

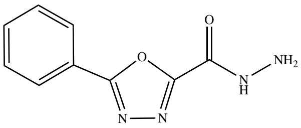

3b

Solvent: DMSO-d6

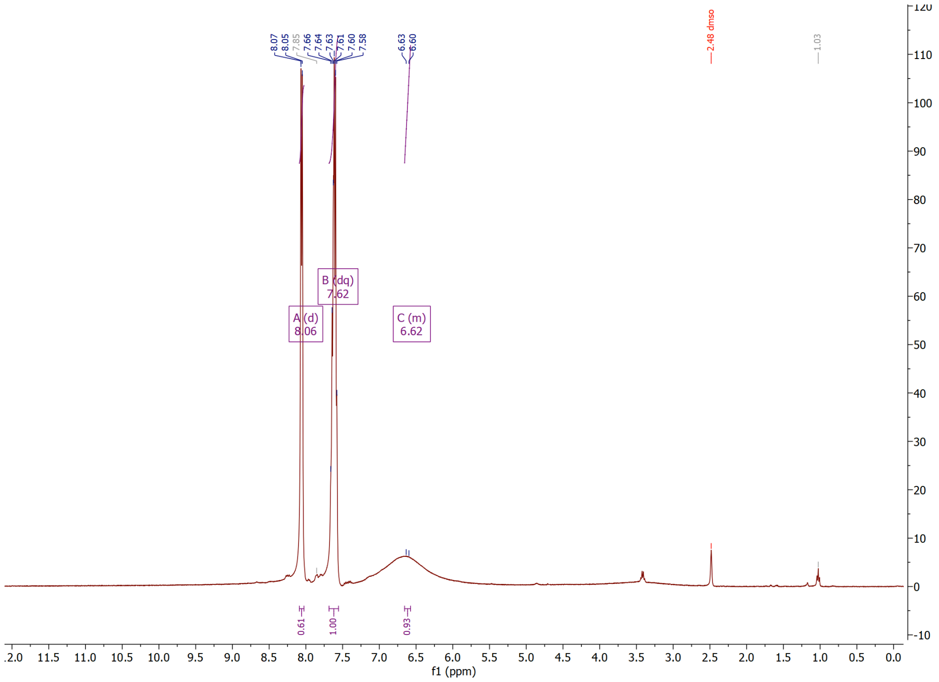

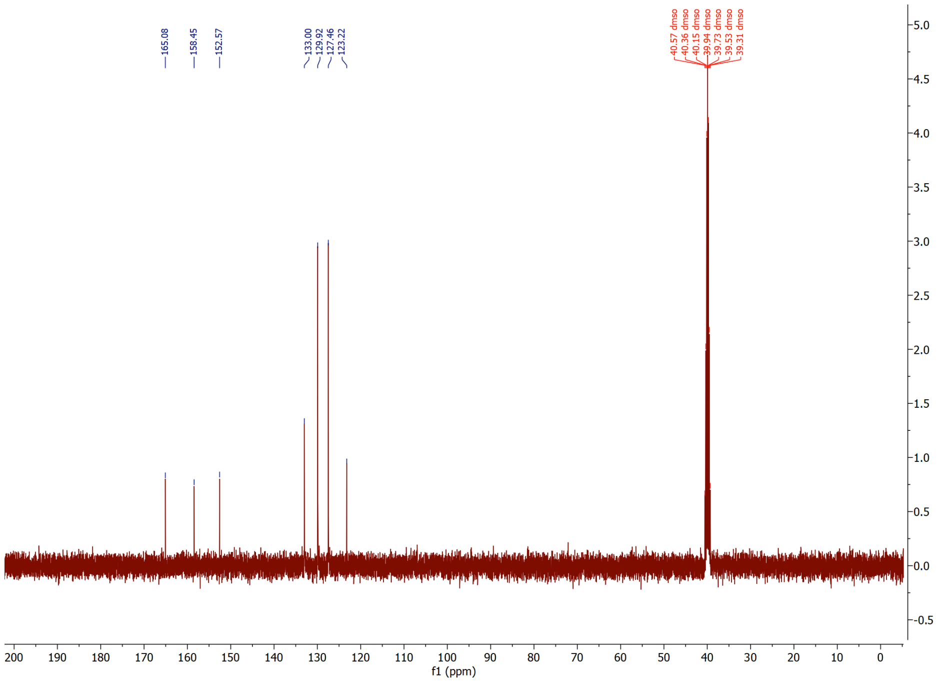

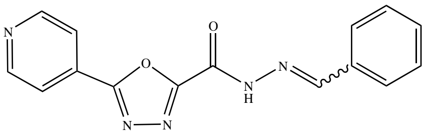

1a

Solvent: DMSO-d6

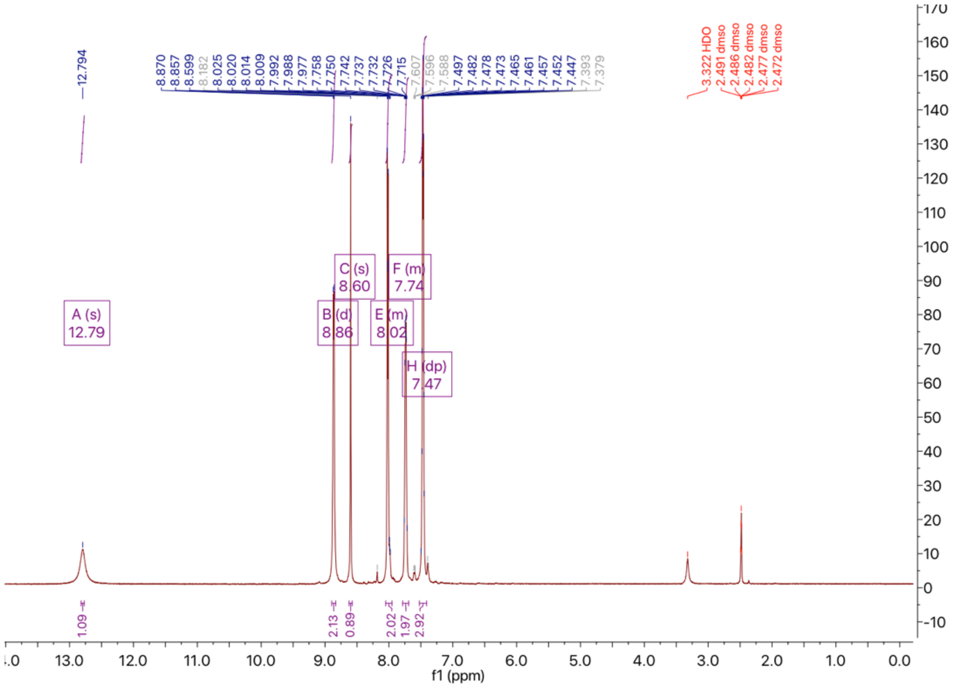

Solvent: DMSO-d6

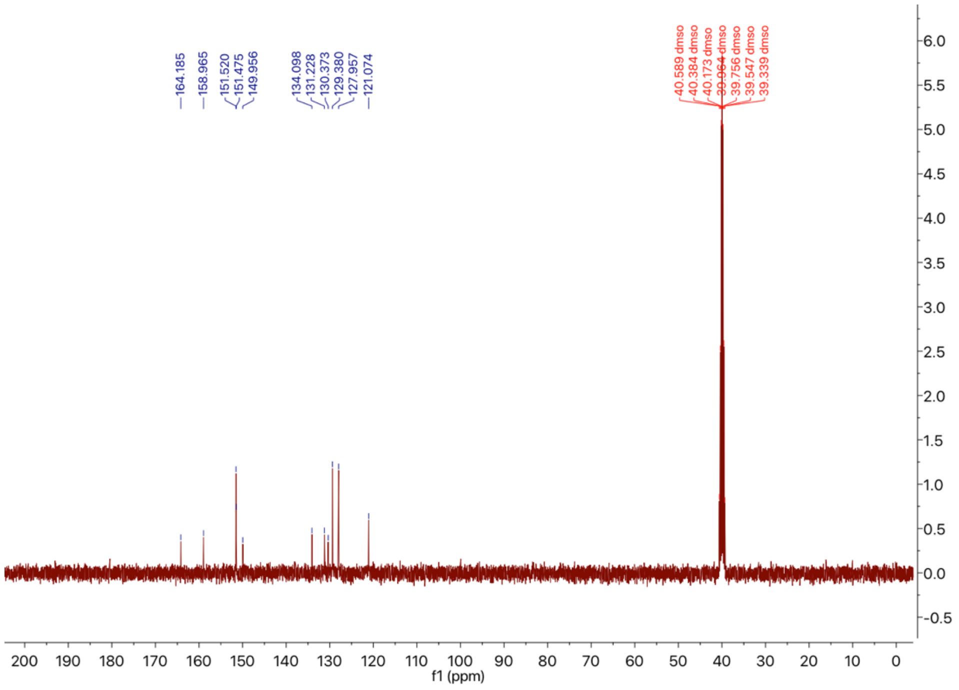

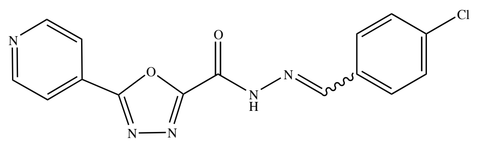

1b

Solvent: DMSO-d6

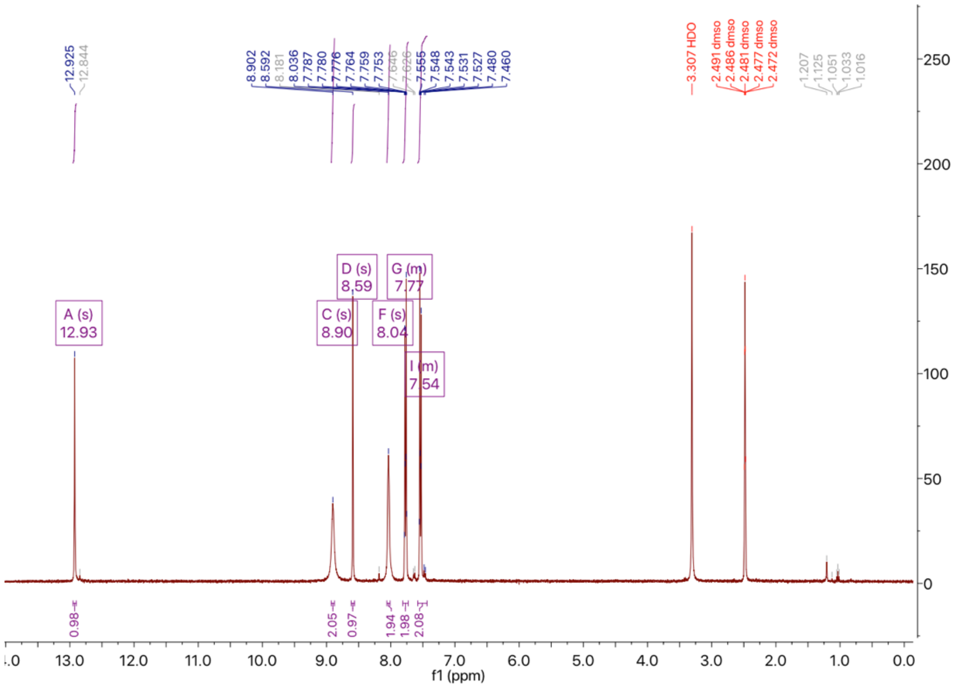

Solvent: C_5D_5N

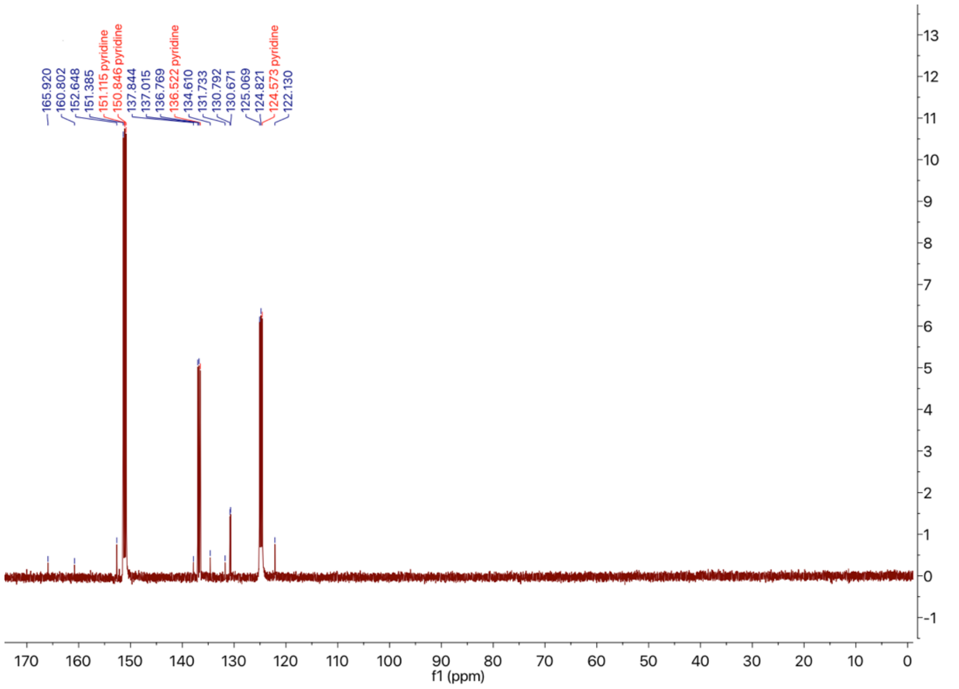

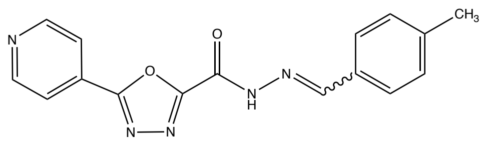

1c

Solvent: $CDCl_3$

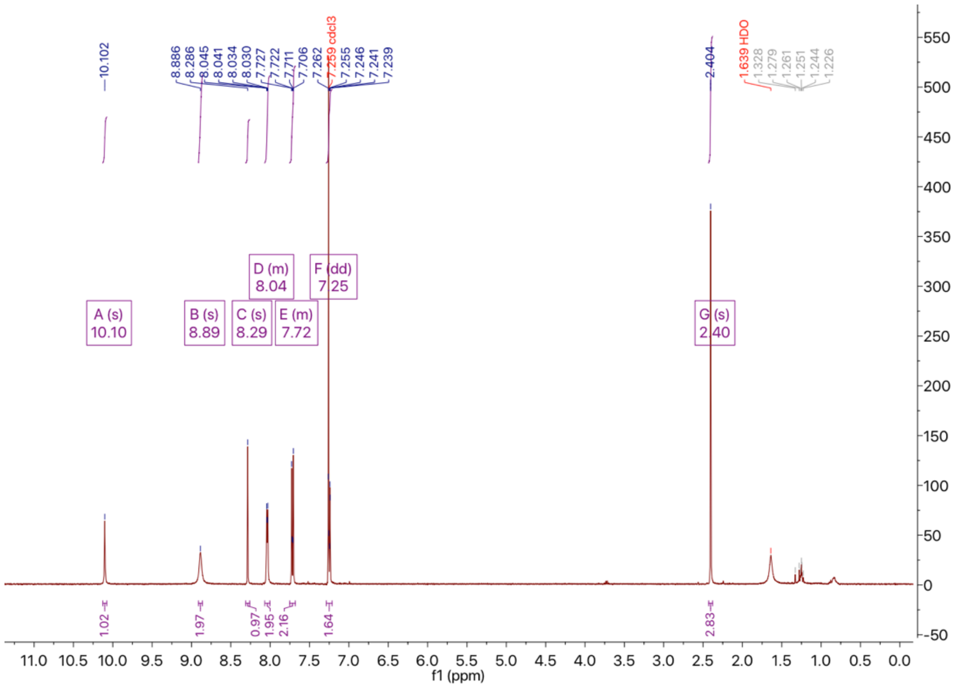

Solvent: $CDCl_3$

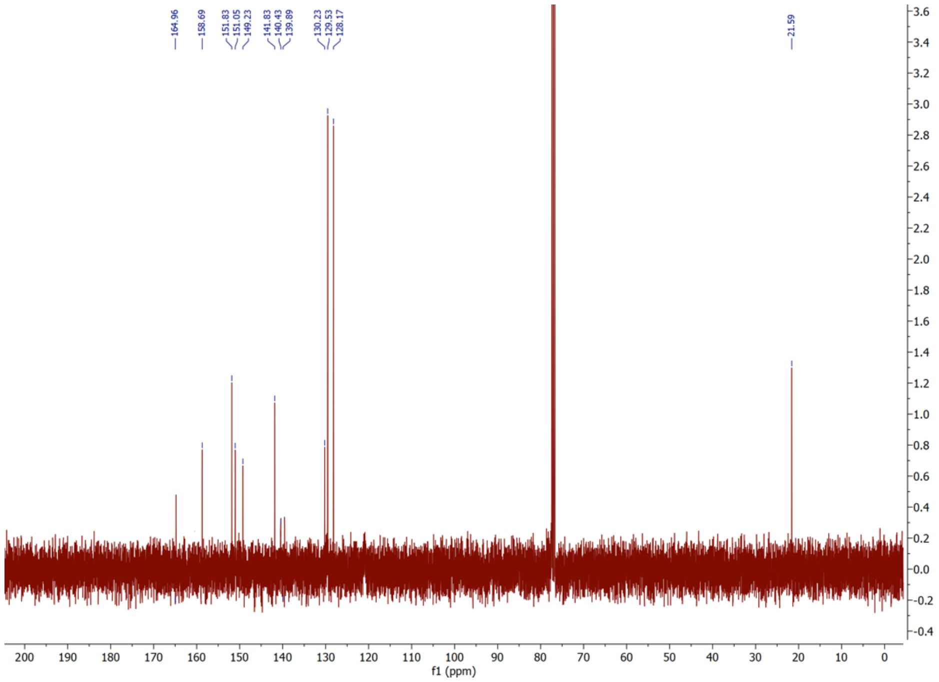

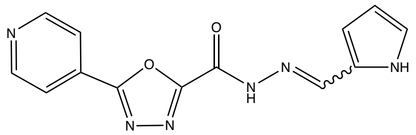

1d

Solvent: DMSO-d6

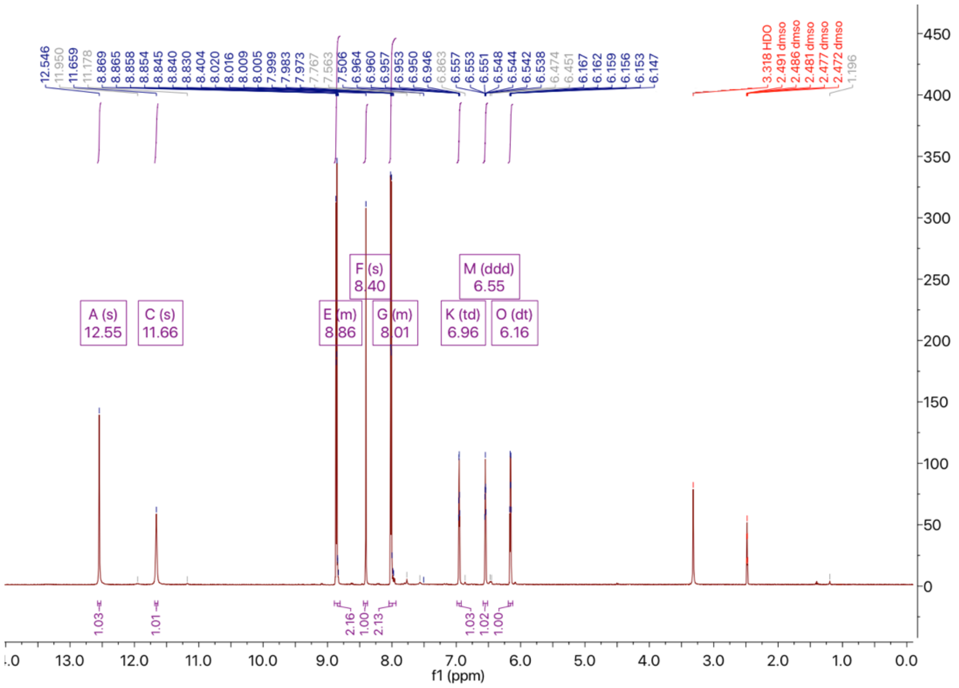

Solvent: DMSO-d6

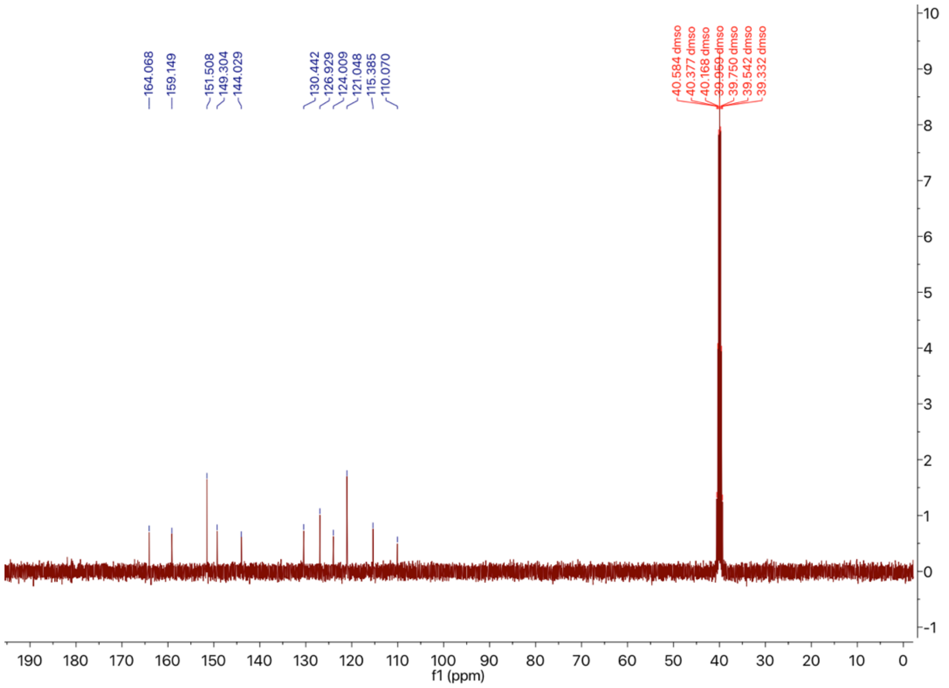

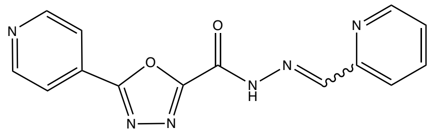

1e

Solvent: DSMO-d6

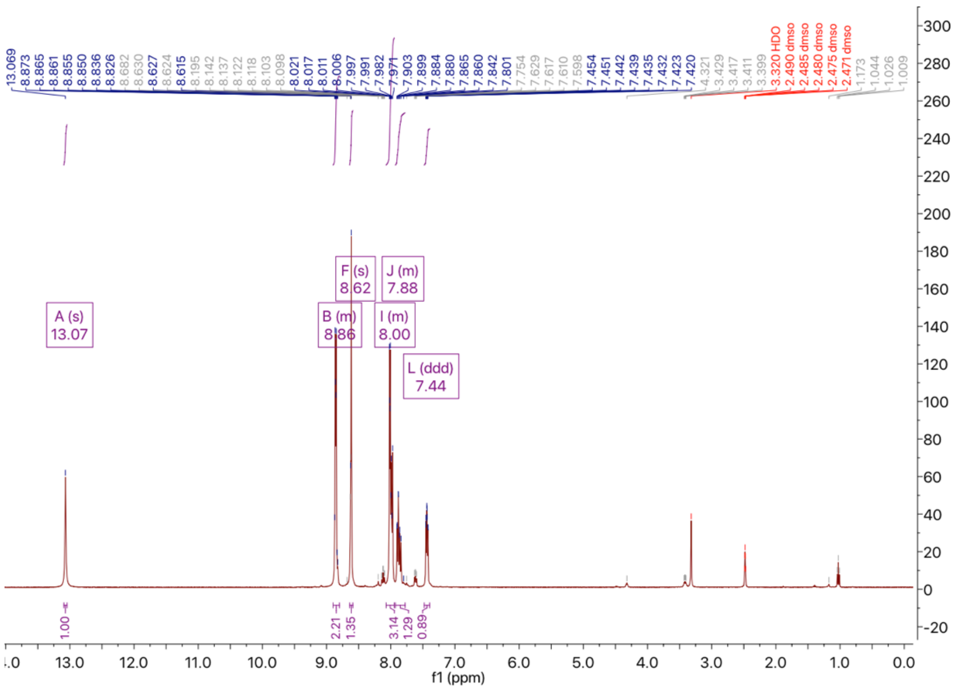

Solvent: DSMO-d6

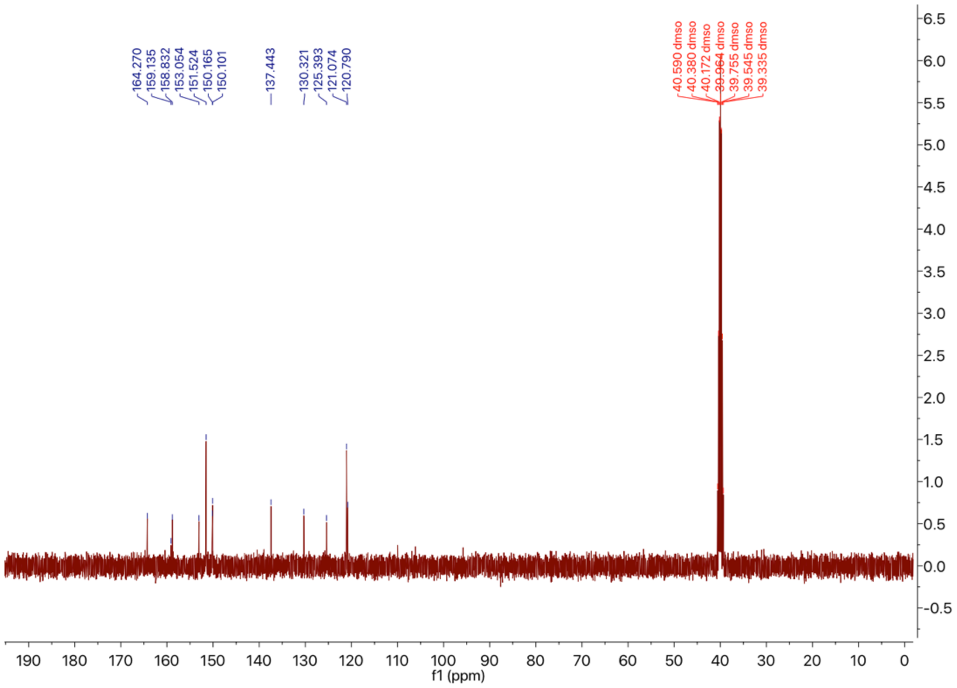

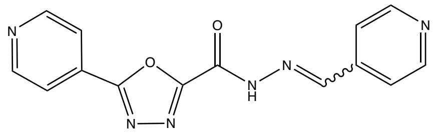

1f

Solvent: DMSO-d6

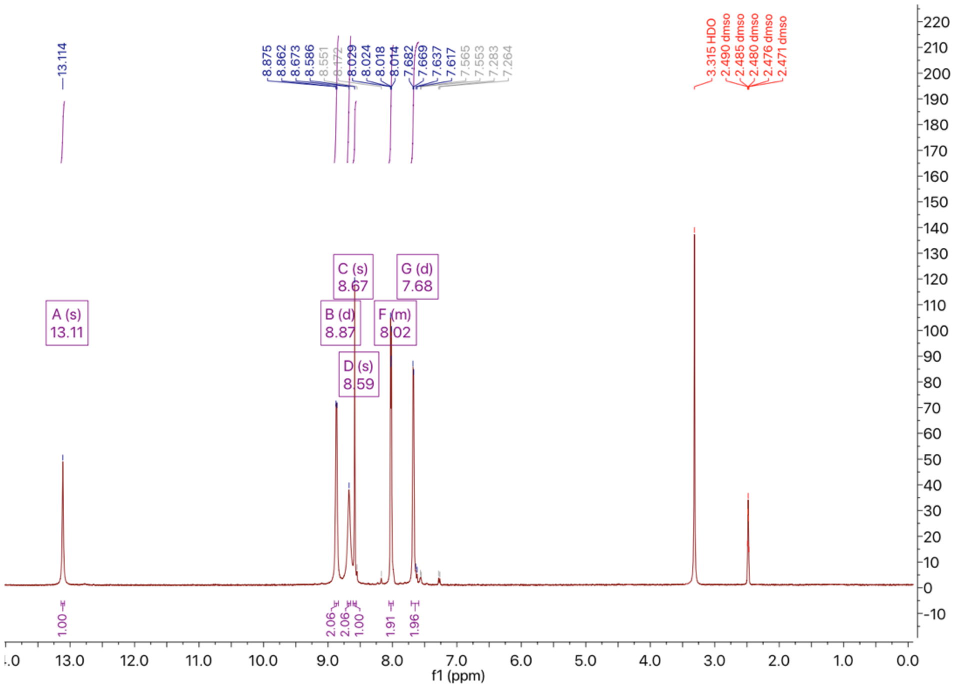

Solvent: DMSO-d6

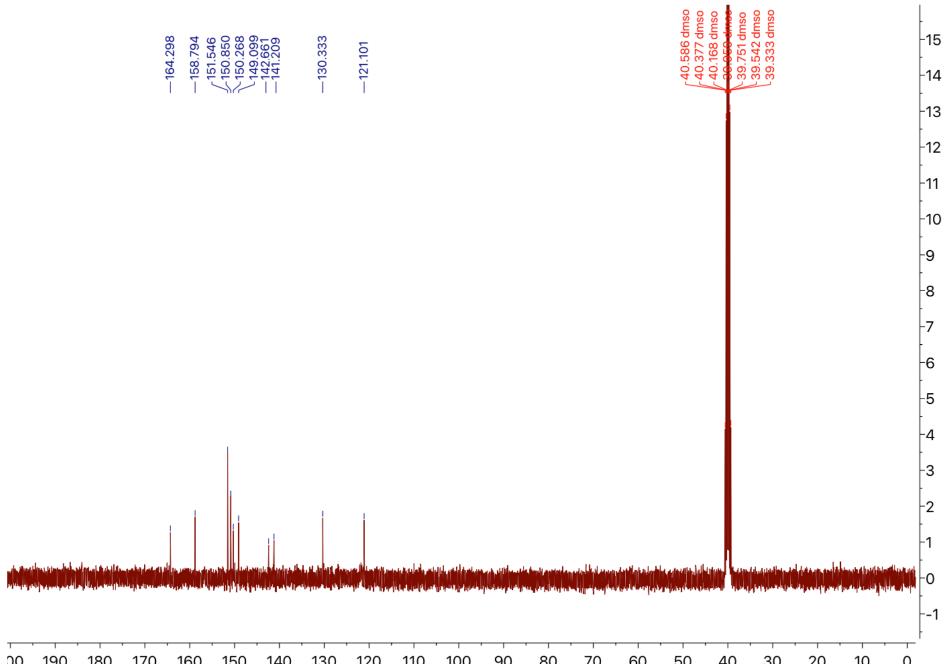

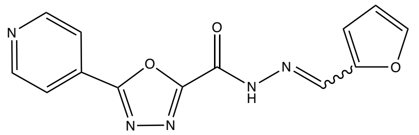

1g

Solvent: DMSO-d6

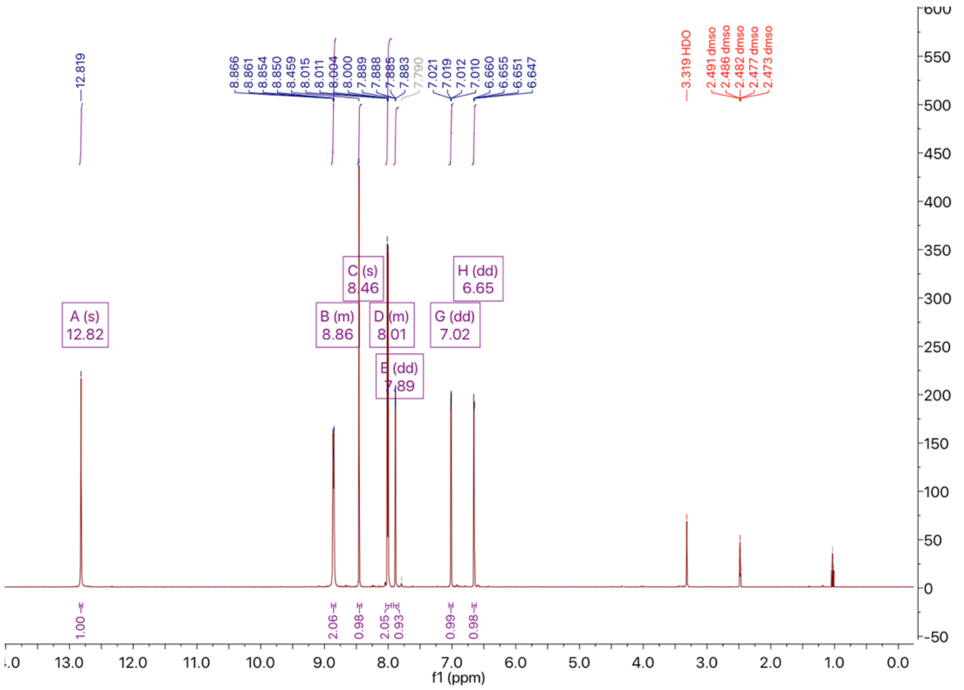

Solvent: DMSO-d6

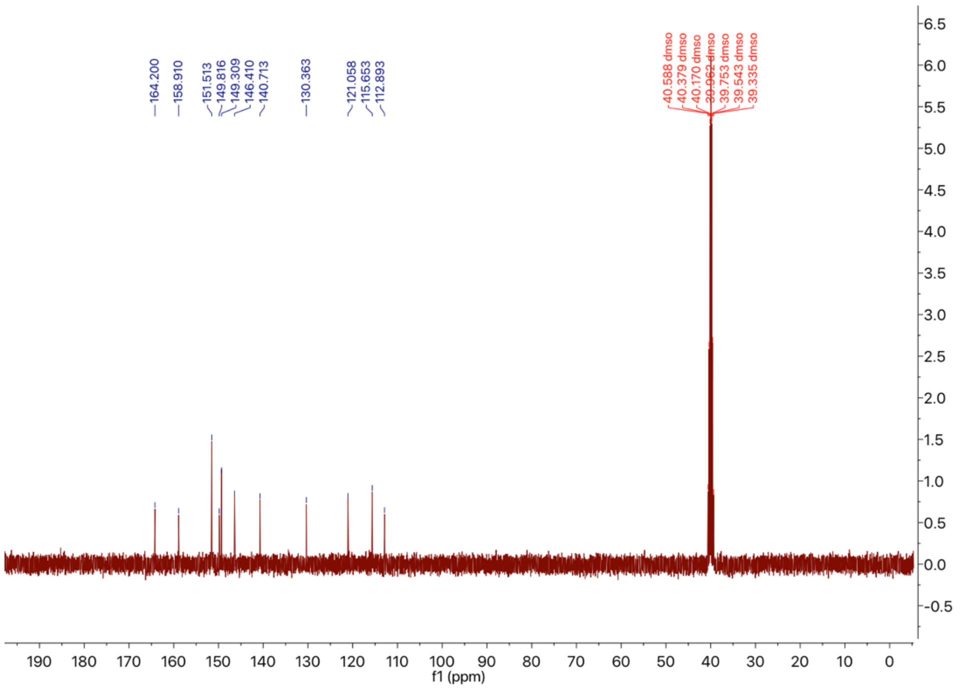

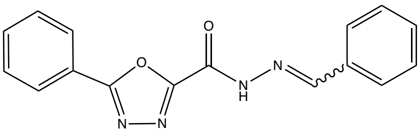

1h

Solvent: DMSO-d6

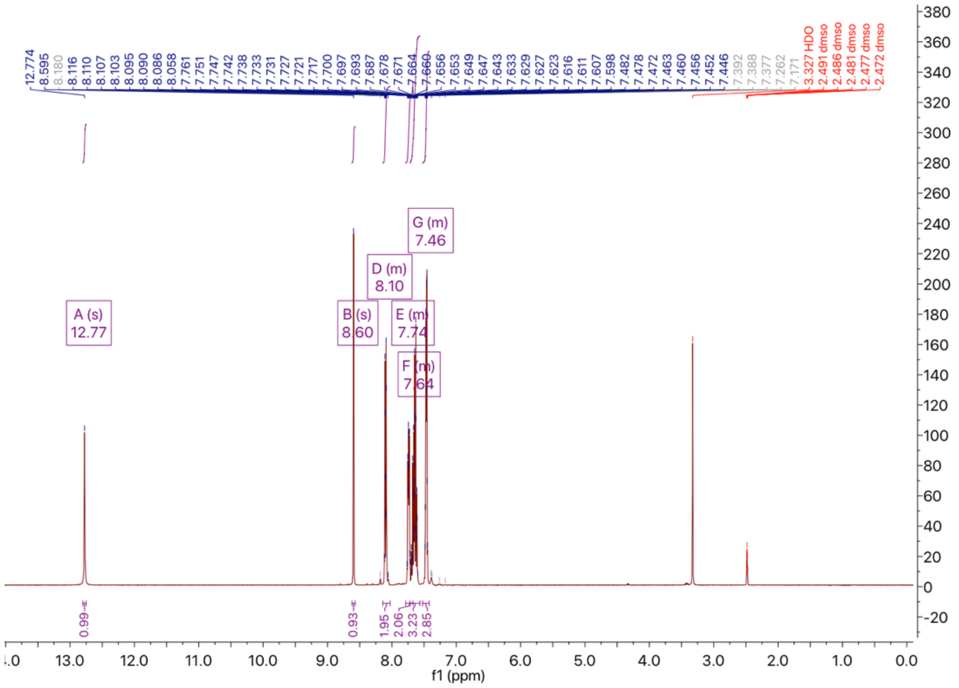

Solvent: DMSO-d6

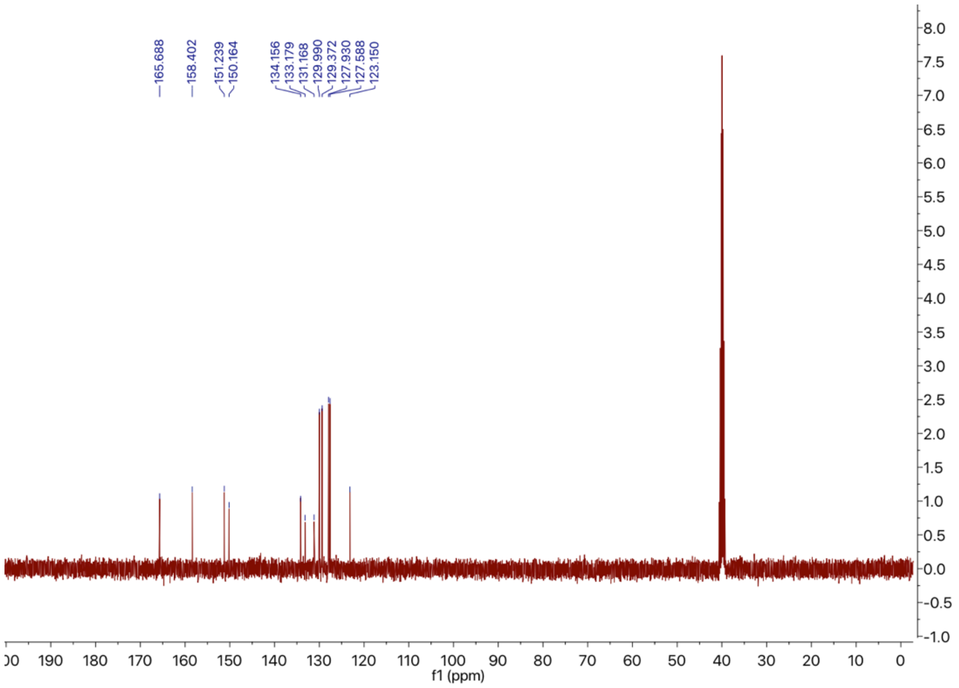

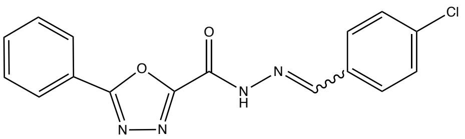

1i

Solvent: DMF-d7

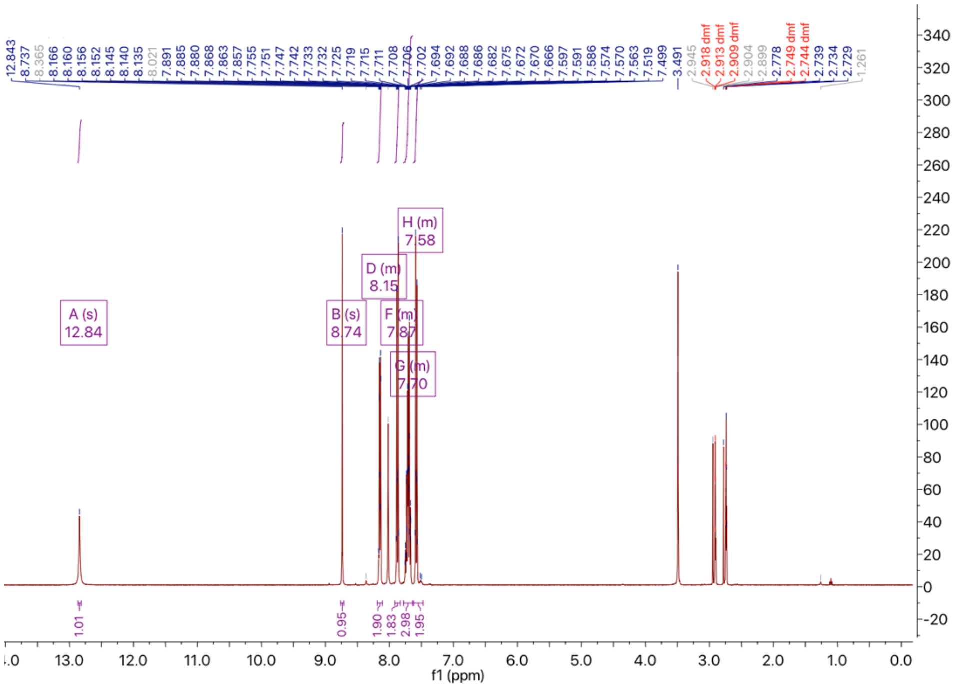

Solvent: DMF-d7

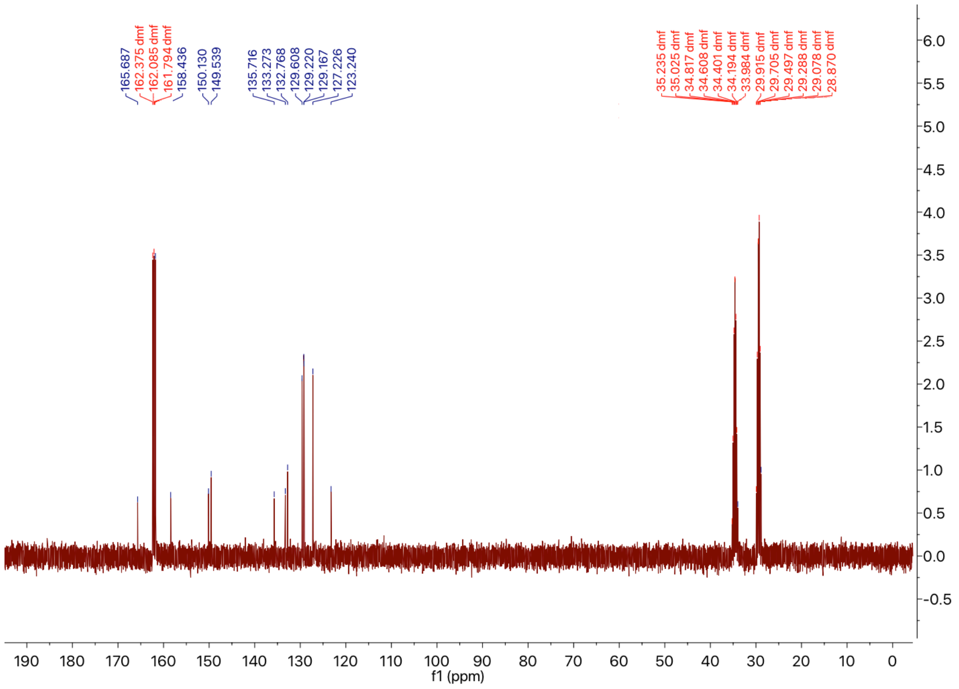

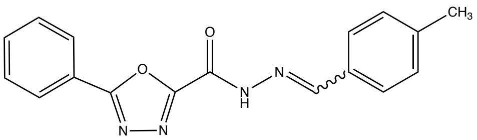

1j

Solvent: DMSO-d6

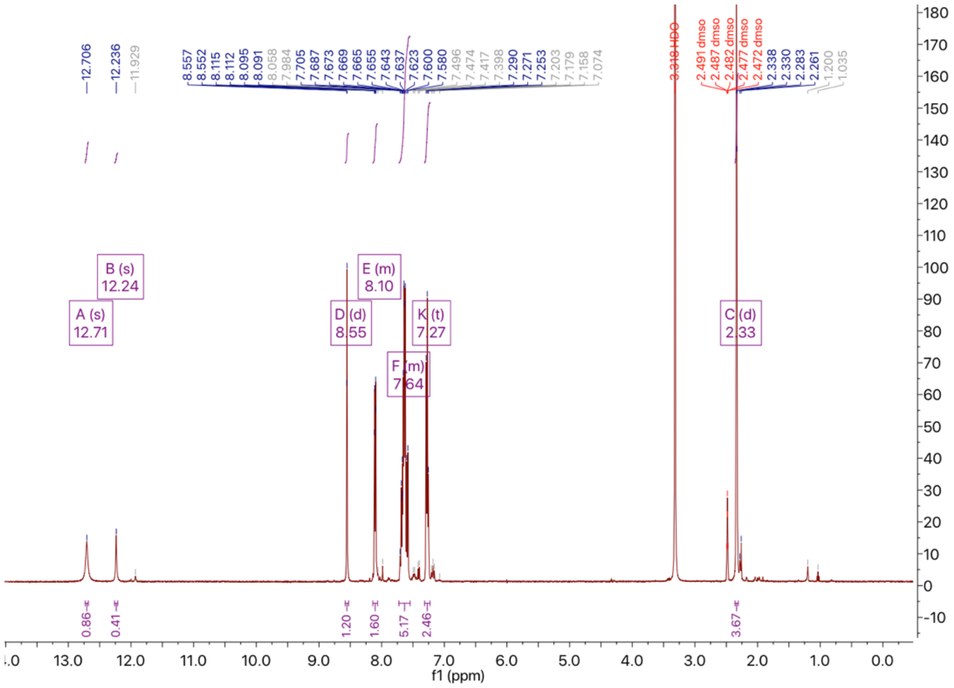

Solvent: DMSO-d6

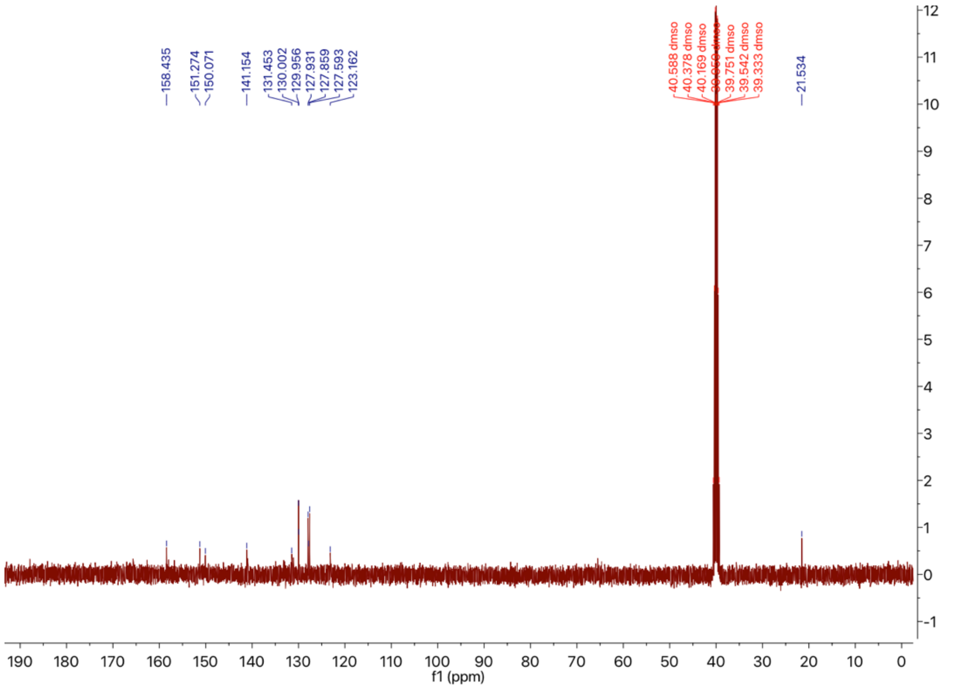

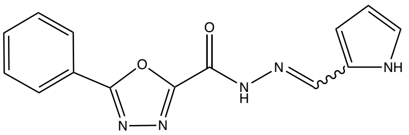

1k

Solvent: DMSO-d6

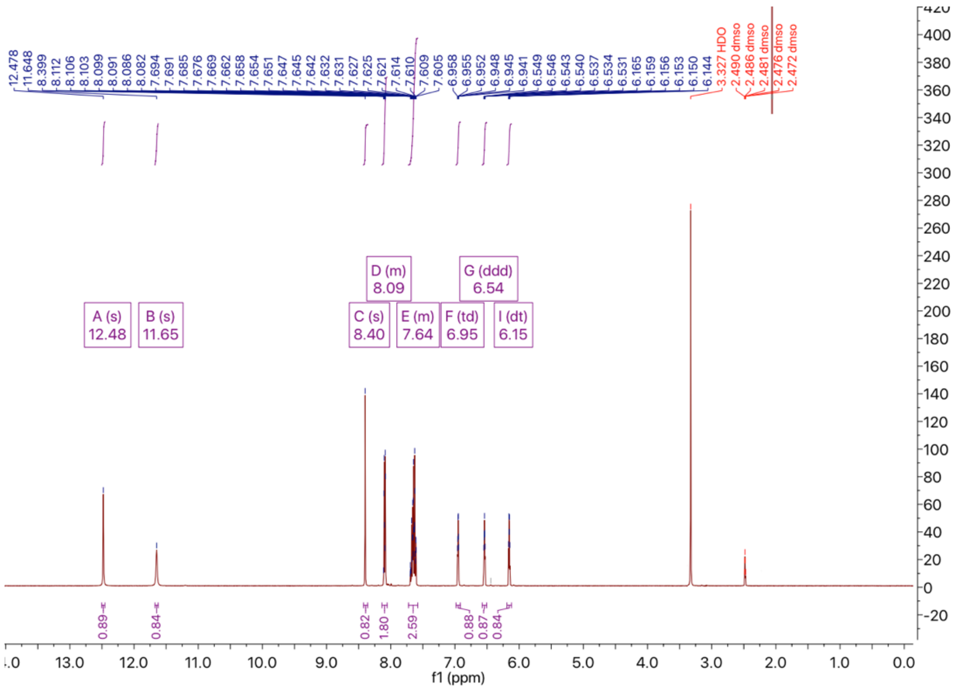

Solvent: DMSO-d6

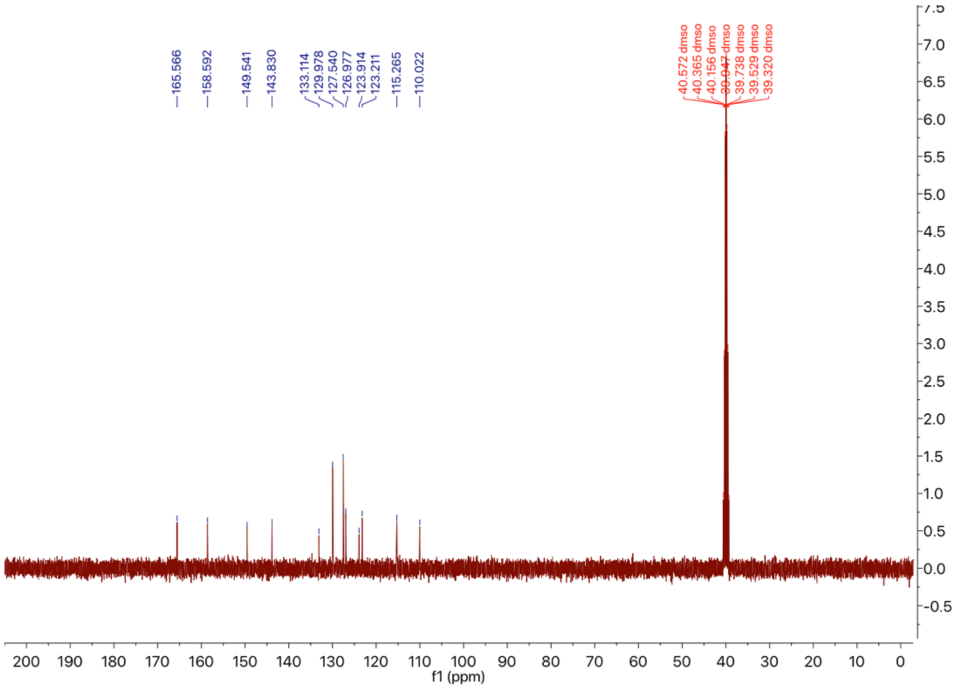

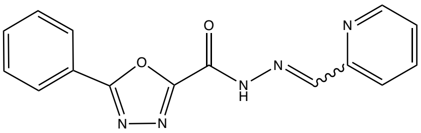

1l

Solvent: DMSO-d6

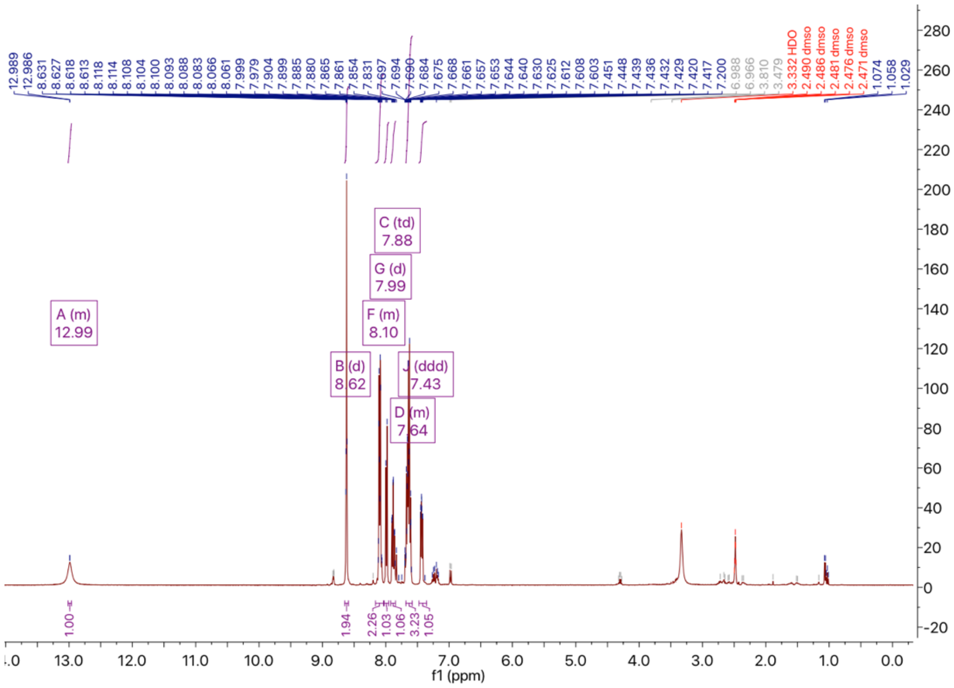

Solvent: DMSO-d6

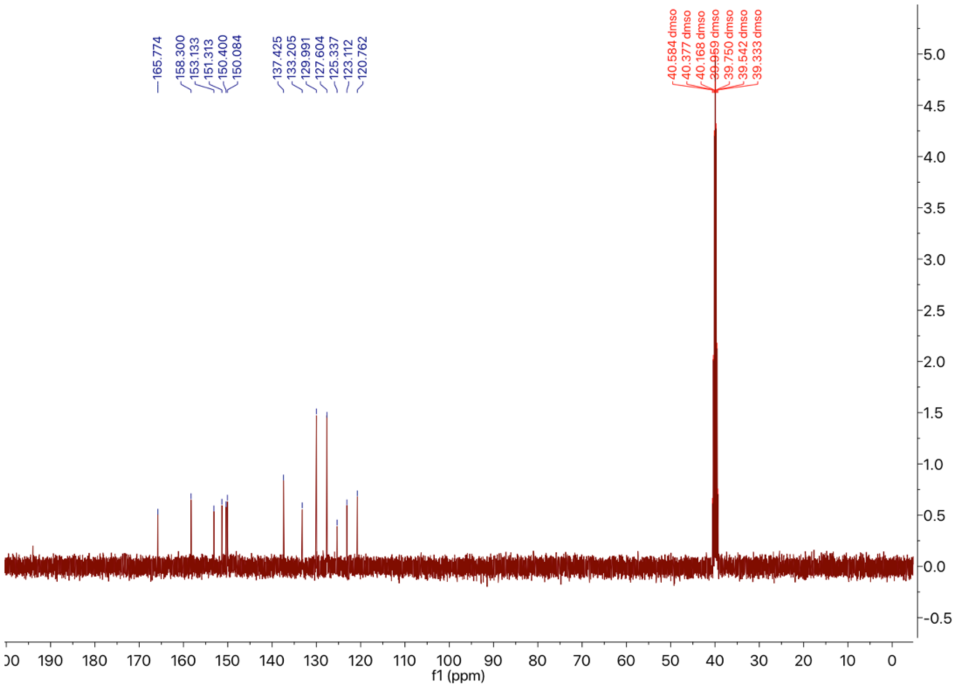

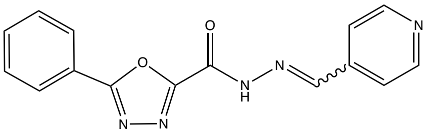

1m

Solvent: DMSO-d6

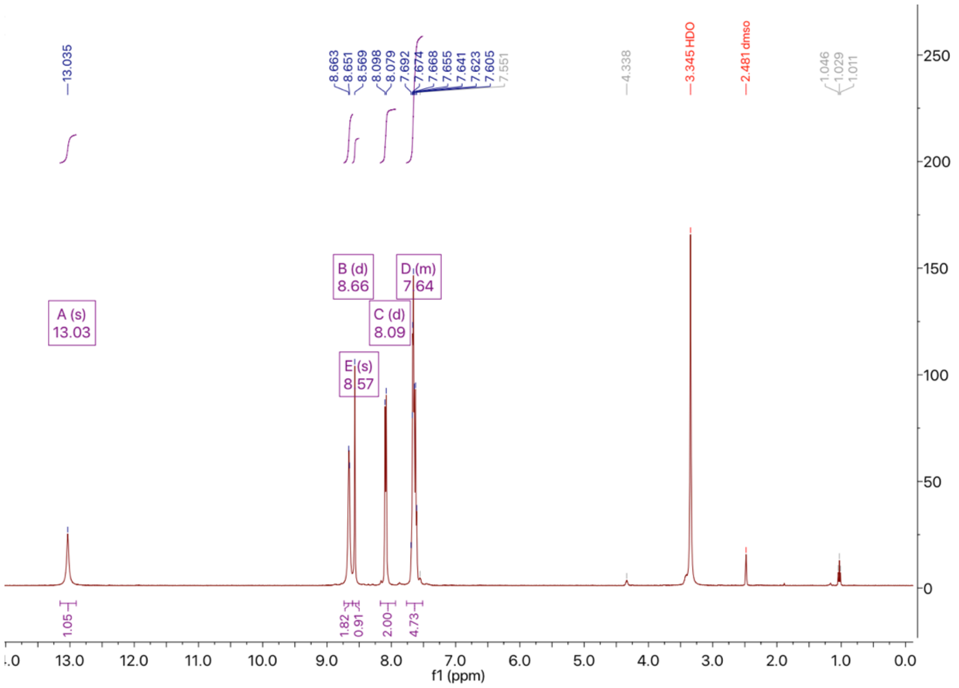

Solvent: DMSO-d6

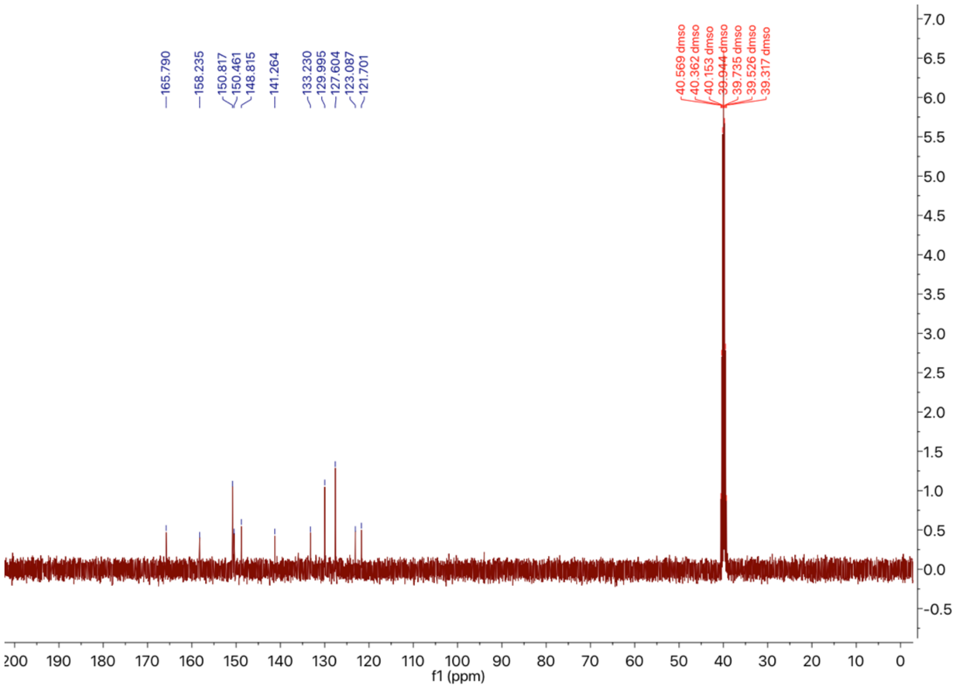

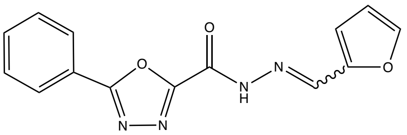

1n

Solvent: DMSO-d6

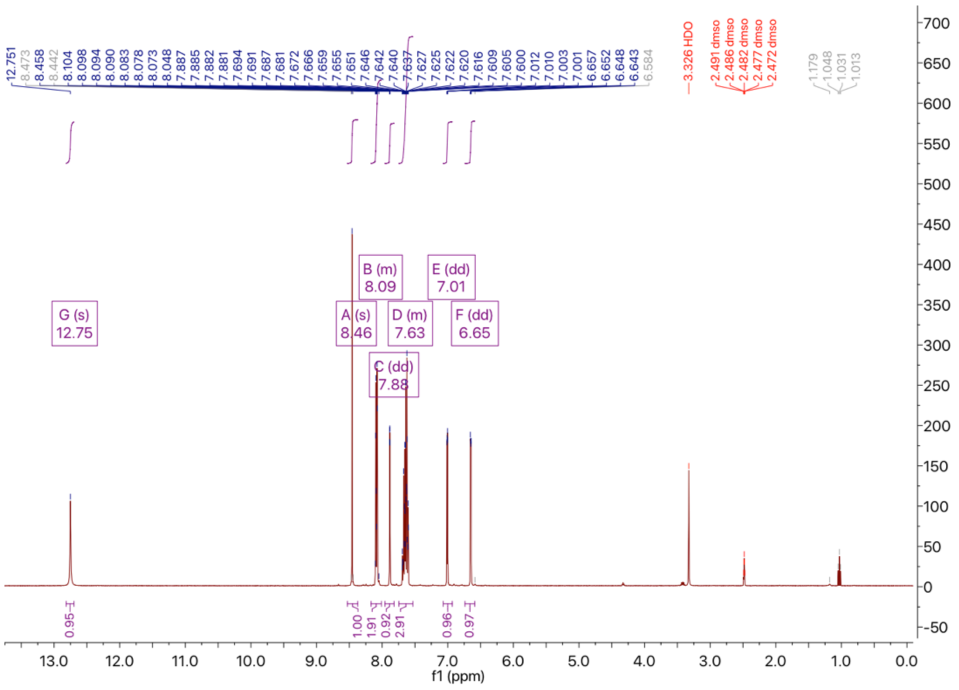

Solvent: DMSO-d6

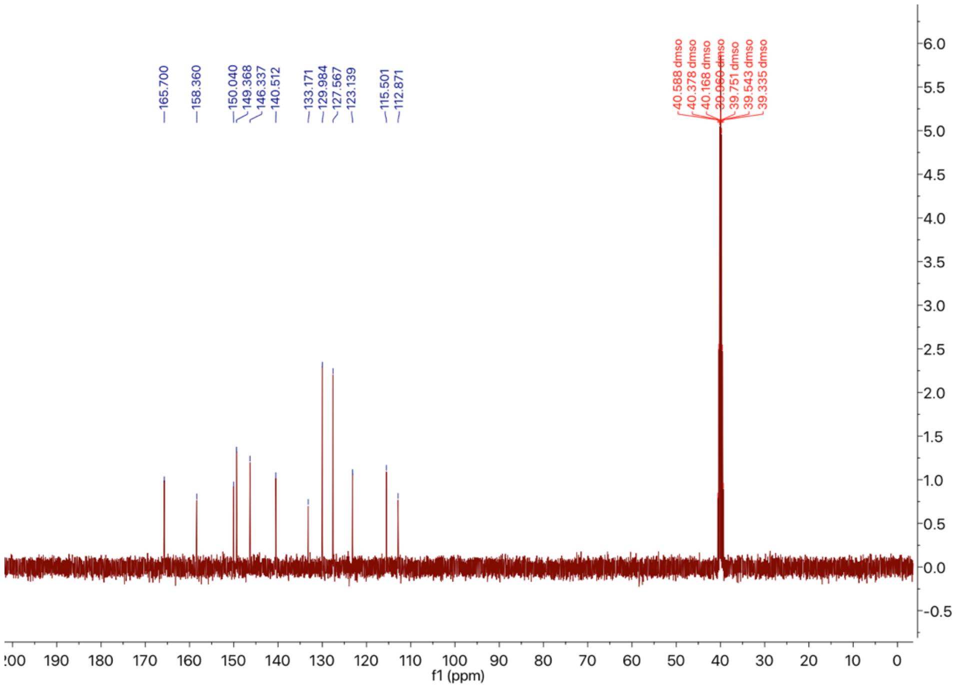

Cytotoxicity study (additional information)

Table S1: *Percent of viability for compounds **1h, 1k–n** vs DMSO in SH-SY5Y cell line.*
The human neuroblastoma cell line SH-SY5Y was treated with 6 different concentrations of the indicated compounds as described in the paragraph 4.2.4. of the main text. Data represent average values ± SEM for triplicate measurements from two independent experiments.

Conc (μM)	Viability (%)				
	**1h**	**1k**	**1l**	**1m**	**1n**
100	97 ± 12	37 ± 6	49 ± 7	61 ± 9	97 ± 11
50	100 ± 14	49 ± 7	70 ± 10	87 ± 11	100 ± 13
25	99 ± 15	81 ± 12	93 ± 13	96 ± 14	100 ± 12
12.5	99 ± 11	101 ± 14	98 ± 13	98 ± 16	99 ± 15
6.25	99 ± 13	100 ± 13	100 ± 12	100 ± 14	100 ± 11
3.125	100 ± 13	100 ± 13	100 ± 12	100 ± 14	100 ± 11

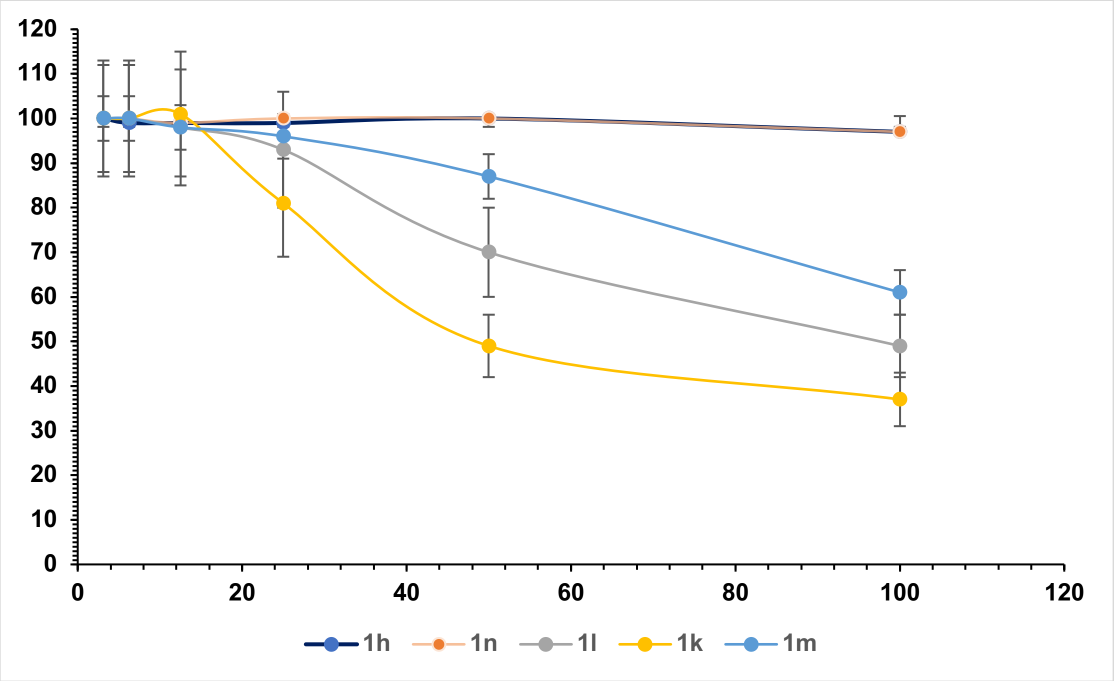

Figure S1: Percentage of viability of tested compounds ± SEM

CC_{50} were determined by curve-fitting.

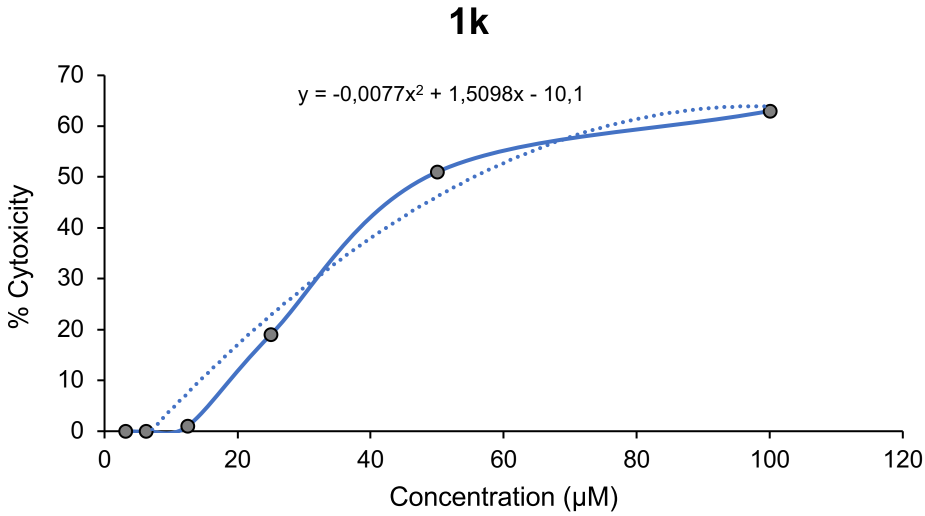

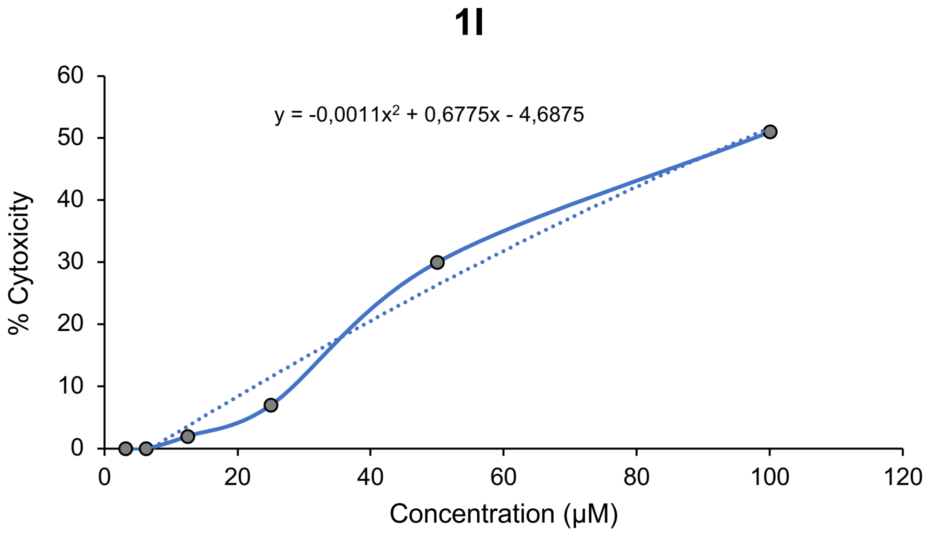

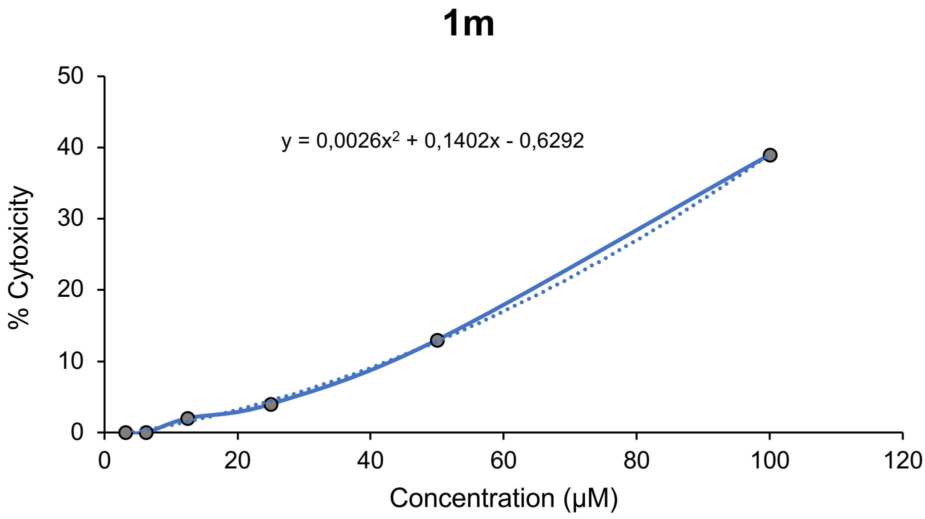

Figure S2-S4: CC_{50} curves for compounds **1k-m**.

Table S2: *CC_{50} (μM) of tested compounds **1h**, **1k–n** in SH-SY5Y cell line.*

Cmpd	CC_{50} (μM)
**1h**	Not observed up to 100
**1k**	50 ± 6
**1l**	100 ± 12
**1m**	128 ± 16
**1n**	Not observed up to 100
